# An Enhanced Numerical Calculation Method to Study the Anchorage Performance of Rebars

**DOI:** 10.3390/ma17163987

**Published:** 2024-08-11

**Authors:** Jianhang Chen, Junming Ma, Xiaofan Zeng, Banquan Zeng, Krzysztof Skrzypkowski, Krzysztof Zagórski, Anna Zagórska, Saisai Wu

**Affiliations:** 1 School of Energy and Mining Engineering, China University of Mining and Technology (Beijing), Beijing 100083, China; 2 Faculty of Civil Engineering and Resource Management, AGH University of Krakow, Mickiewicza 30 Av., 30-059 Kraków, Poland; 3 Faculty of Mechanical Engineering and Robotics, AGH University of Krakow, Mickiewicza 30 Av., 30-059 Kraków, Poland; 4 Research Centre in Kraków, Institute of Geological Sciences, Polish Academy of Science, Senacka 1, 31-002 Kraków, Poland; 5 School of Resources Engineering, Shanxi Key Laboratory of Geotechnical and Underground Space Engineering, Xi’an University of Architecture & Technology (XAUAT), Xi’an 710055, China

**Keywords:** numerical calculation, anchorage performance, rebar–grout interface, peak load, softening section

## Abstract

When modelling the anchorage performance of rebars with the tri-linear law, the calculation process of the load–deformation relation is complicated. The reason is that when the rebar–grout interface entered the elastic–softening–debonding stage, the softening section length and debonding section length vary simultaneously. To solve this issue, this paper proposes an enhanced numerical calculation method. When the rebar–grout interface entered the elastic–softening–debonding stage, the softening section length was fixed to a specific value. One loop function was created to calculate the debonding section length. With this method, the number of iteration calculations significantly decreased. The credibility of this calculation method was confirmed with experimental results. Two case studies were conducted to compare the load–deformation relation obtained with the original calculation method and enhanced calculation method. The results showed that good consistency existed between the results obtained by those two methods. This finding can significantly improve the calculation efficiency when studying the anchorage performance of rebars. Moreover, this paper provides new insight for users to optimise the modelling process of rebars.

## 1. Introduction

Rebars are commonly used in civil and mining engineering [1,2,3,4]. In civil engineering, they are used to reinforce concrete blocks. The purpose of adding rebars to concrete blocks is to increase the tensile capacity of reinforced concrete blocks. In mining engineering, rebars are crucial in improving the integrity and stability of rock masses [5,6,7]. The reason is that in mining engineering, the rock mass is typically composed of rock joints [8,9,10]. Because of these rock joints, the strength of rock masses is usually weak [11,12,13]. Therefore, once excavation is conducted in mine sites, rock mass collapsing may occur, leading to geological disasters [4]. Engineering practices demonstrate that when rebars are used to reinforce rock masses around an excavation, the stability of jointed rock masses can be significantly improved [5].

After rebars are installed in concrete blocks or rock masses, the deformation of concrete blocks or rock masses induces tensile elongation of rebars [14]. This further leads to load transfer between concrete blocks, rock masses, and rebars via grout [15,16]. The process directly represents the anchorage performance of rebars. To study anchorage performance rebars, researchers and engineers have proposed massive analytical models [17].

Yazici and Kaiser [18] proposed a contact model to predict the peak load of rock reinforcement tendons. The uneven surface of rock reinforcement tendons was assumed to be the zigzag profile. Then, a rock joint equation was used to analyse the interaction between rock reinforcement tendons and grout. Moreover, this model was further improved to study the performance of rock tendons when the ambient stress changed. Hyett et al. [19] conducted similar research to obtain the load–deformation relation of rock reinforcement tendons. However, they spent more effort on the whole load–deformation relation. Cao et al. [20] focused on the surface geometry of rebars. They analysed the rib geometry along the tensile loading direction of rebars, including rib spacing, rib height, and width. They believed that the rib geometry could cut the resin grout along different angles. Based on this concept, they developed an analytical model to predict the anchorage capacity of rebars. Cao et al. [21] further calculated the Poisson influence on the tensile performance of rebars. They used experimental push tests and pull tests to confirm the credibility of this model. Cao et al. [22] optimised the selection method of rebars to prevent the bond failure of the rock reinforcement system. Specifically, they developed an analytical model to calculate the anchorage capacity of rebars being subjected to bond failure. Ma et al. [23] analysed the bond failure at the rebar–grout interface. The yielding behaviour of rebars was considered. Reasonable agreement existed between modelling and experimental results. Li et al. [24] found that in the rock reinforcement system, cone shape failure mode may occur. Aiming at revealing the corresponding failure mechanism, they developed an analytical model. The degradation process of the grout was analysed. Based on this degradation process, they successfully simulated the load–deformation relation of rock reinforcement tendons.

The above research makes a significant contribution to revealing the anchorage mechanism of rebars. However, a common shortcoming is that more attention was paid to short encapsulating rebars. In this case, the inhomogeneous shear stress (SS) distribution at the rebar–grout interface was neglected. Considering this issue, Farmer [25] analysed the SS distribution at the rebar–grout interface and proposed an analytical model to simulate this SS distribution. However, a shortcoming is that his model is applicable for scenario where rebars are permanently bonded with the confining medium. This shortcoming also occurred in other similar research [26].

To overcome this shortcoming, other researchers tried to simulate the bonding and debonding behaviour of the rebar–grout interface. To realise this, the bonding–deformation law of the rebar–grout interface should be determined. Several bonding–deformation laws have been proposed. Initially, the tri-linear law was adopted to analyse the shear behaviour of the rebar–grout interface. Later, this tri-linear law was confirmed and validated by many researchers. Cai et al. [27] developed a bi-linear law. This law was successfully used to predict the anchorage performance of rebars reinforced in soft rock tunnels. Moreover, this bilinear law was also applied in predicting the anchorage performance of rebars in underground mines [28]. Ma et al. [29] proposed a closed non-linear law to analyse the shear behaviour of the rebar–grout interface. A similar non-linear law was also used in other research [30].

Among those mentioned bonding–deformation laws, the tri-linear law is more widely accepted. The reason is that this tri-linear law can consider the SS increasing, SS decreasing, and debonding behaviour of the rebar–grout interface. Moreover, after this tri-linear law was incorporated into the reinforcement system, it solved those mathematical equations. Nevertheless, the tri-linear law still has shortcomings. Specifically, when the tri-linear law is used, the rebar–grout interface will experience the elastic–softening–debonding (ESD) stage. In this stage, the length of the softening section and debonding section are mobilised during this whole stage. This makes the solving process quite complicated.

To solve this issue, this paper proposes an enhanced calculation method. Based on this enhanced calculation method, the load–deformation relation of rebars can be easily simulated. In this paper, first, the decoupling behaviour of the rebar–grout interface is explained. Then, this enhanced calculation method is illustrated. Experimental tests were used to validate this enhanced calculation method. Then, case studies are provided to compare the enhanced calculation method with the original calculation method. Last, a discussion is provided.

## 2. Debonding Process of the Rebar–Grout Interface

**Figure 1:**
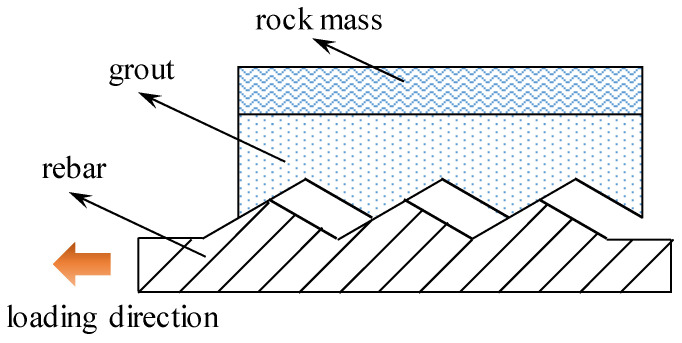
Geometry of the rebar at the rebar–grout interface.

Rebars have threaded geometry at the surface. The function of those threads is to increase the friction and interlocking of the rebar–grout interface [31]. To simulate the rebar surface geometry (Figure 1), previous research assumed that rebars had a zigzag profile [18].

Compared with smooth bars, the bond strength of the rebar–grout interface is significantly improved [32]. Wang et al. [33] indicated that the rebar–grout interface had apparent bond strength and residual bond strength. To simulate the bond strength and residual bond strength, this paper adopted the tri-linear law. The SS and the shear slipping of the rebar–grout interface are illustrated with Equation (1):

(1) τ=ks+b

where *τ* is the SS at the rebar–grout interface; *k* is the slope between the SS and shear slipping; *s* is the shear slipping of the rebar–grout interface; and *b* is a constant coefficient.

Then, this tri-linear law will be merged into the rock reinforcement system to study the interaction between rebars and grout annulus. For fully grouted rebars, the primary failure type was the bond failure at the rebar–grout interface [34]. Therefore, this paper focused on the bond failure at the rebar–grout interface. As for the rock mass, it was assumed to be homogeneous.

## 3. The Enhanced Calculation Method

When the tri-linear law is used to study the shear behaviour of the rebar–grout interface, the whole load–deformation relation of rebars is composed of five stages. They are the elastic stage, the elastic–softening (ES) stage, the elastic–softening–debonding (ESD) stage, the softening–debonding stage, and the debonding stage. This paper followed the deduction procedures used by Ren et al. [35]. When the rebar–grout interface deforms elastically, Equation (2) is the governing equation for studying the anchorage performance of rebars:

(2) $$\frac{d^{2}u}{dx^{2}}-\alpha^{2}u=0$$

where *u* is the tensile displacement of rebars and *α* is a variable coefficient, which can be expressed as Equation (3):

(3) $$\alpha^{2}=\frac{2\tau_{1}}{u_{1}rE}$$

where *τ*_1_ is the bond strength of the rebar–grout interface; *u*_1_ is the shear slipping when the bond strength is reached; *r* is the radius of rebars; and *E* is the modulus of rebars.

When the rebar–grout interface softens, Equation (4) is the governing equation to study the anchorage performance of rebars:

(4) $$\frac{d^{2}u}{dx^{2}}+\omega^{2}u-\frac{2\left(\tau_{1}u_{2}-\tau_{2}u_{1}\right)}{rE\left(u_{2}-u_{1}\right)}=0$$

where *τ*_2_ is the residual bond strength of the rebar–grout interface; *u*_2_ is the shear slipping when the residual bond strength is reached; *ω* is a variable coefficient and can be expressed with Equation (5):

(5) $$\omega^{2}=\frac{2\left(\tau_{1}-\tau_{2}\right)}{rE\left(u_{2}-u_{1}\right)}$$

When the rebar–grout interface debonds, Equation (6) is the governing equation to study the anchorage performance of rebars.

(6) $$\frac{d^{2}u}{dx^{2}}=\frac{2\tau_{2}}{rE}$$

Based on Equations (2)–(6), the whole load–deformation relation of rebars can be simulated.

Specifically, for the elastic stage, the load–deformation relation can be expressed as Equation (7):

(7) $$p=AE\alpha \tanh\left(\alpha l\right)u$$

where *p* is the tensile load of rebars; *A* is the cross-sectional area of rebars; and *l* is the encapsulating length of rebars.

Then, for the ES stage, the load–deformation relation can be expressed with Equations (8) and (9):

(8) $$u=\frac{2\tau_{1}}{rE\omega }\left(\frac{\tanh\left(\alpha \left(l-l_{s}\right)\right)\sin\left(\omega l_{s}\right)}{\alpha }-\frac{\cos\left(\omega l_{s}\right)}{\omega }\right)+\frac{\tau_{1}u_{2}-\tau_{2}u_{1}}{\tau_{1}-\tau_{2}}$$

(9) $$p=c\tau_{1}\left(\frac{\tanh\left(\alpha \left(l-l_{s}\right)\right)\cos\left(\omega l_{s}\right)}{\alpha }+\frac{\sin\left(\omega l_{s}\right)}{\omega }\right)$$

where *l_s_* is the length of the softening section and *c* is the perimeter of rebars.

The length of the softening section increases from zero. When the ES stage ends, the length of the softening section reaches the first critical value (*l_s_*__*cri*1_), which can be expressed with Equation (10):

(10) $$l_{s\_cri1}=\frac{1}{\omega }\left(\arcsin\frac{\alpha }{\sqrt{\alpha^{2}+\omega^{2}}}-\arcsin\frac{\tau_{2}\alpha }{\tau_{1}\sqrt{\alpha^{2}+\omega^{2}}}\right)$$

Finally, for the ESD stage, the load–deformation relation can be expressed as Equations (11) and (12):

(11) $$u=u_{2}+\frac{1}{rE}\left(\tau_{2}l_{d}^{2}+\frac{2\tau_{1}l_{d}\tanh\left(\alpha \left(l-l_{d}-l_{s}\right)\right)\cos\left(\omega l_{s}\right)}{\alpha }+\frac{2\tau_{p}l_{d}\sin\left(\omega l_{s}\right)}{\omega }\right)$$

(12) $$p=c\tau_{2}l_{d}+c\tau_{1}\left(\frac{\tanh\left(\alpha \left(l-l_{d}-l_{s}\right)\right)\cos\left(\omega l_{s}\right)}{\alpha }+\frac{\sin\left(\omega l_{s}\right)}{\omega }\right)$$

where *l_d_* is the length of the debonding section.

**Figure 2:**
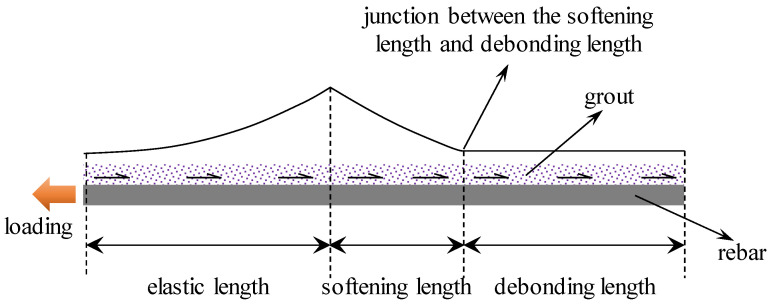
SS distribution in the ESD stage.

In the ESD stage, there is a specific relation between the softening section and the debonding section. Theoretically, at the junction between them, the SS equal the residual shear strength, as shown in Figure 2.

At the junction between the softening section and the debonding section, the SS at the rebar–grout interface can be expressed with Equation (13):

(13) $$\tau =\tau_{1}\cos\left(\omega l_{s}\right)-\frac{\tau_{1}\omega }{\alpha }\tanh\left(\alpha \left(l-l_{d}-l_{s}\right)\right)\sin\left(\omega l_{s}\right)$$

Then, letting Equation (13) equal the residual bond strength, the relation between the softening section and debonding section can be obtained.

When the ESD stage ends, the softening section length reaches the second critical value (*l_s_*__*cri*2_), which can be expressed with Equation (14):

(14) $$l_{s\_cri2}=\frac{1}{\omega }\arccos\frac{\tau_{2}}{\tau_{1}}$$

According to Equations (10) and (14), in the ESD stage, the length of the softening section increases from *l_s_*__*cri*1_ to *l_s_*__*cri*2_. In the meantime, the length of the debonding section increases from zero to (*l*-*l_s_*__*cri*2_).

To obtain the specific length of the softening section and debonding section, Equation (13) should be solved. However, this equation comprises trigonometric functions and the hyperbolic tangent function. It is quite complicated to obtain an accurate analytical solution. To solve Equation (13), numerical calculation can be conducted since numerical calculation is efficient in solving geotechnical issues.

**Figure 3:**
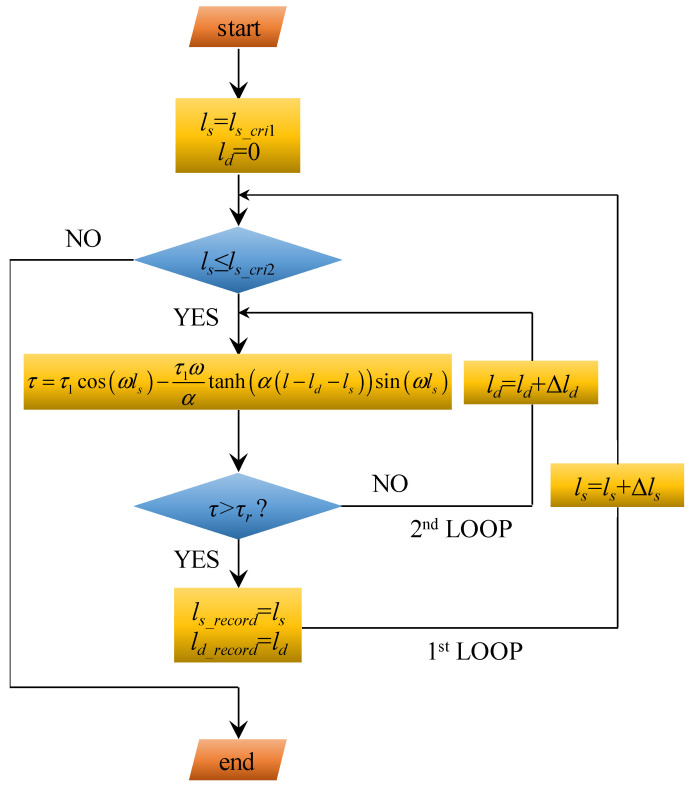
A numerical calculation method to obtain the length of the softening section and debonding section.

To realise this, the numerical calculation method shown in Figure 3 can be used. At the initial state of the ESD stage, the length of the softening section equals *l_s_*__*cri*1_ and the length of the debonding section equals zero. Then, logic was applied to check whether the length of the softening section was not larger than *l_s_*__*cri*2_. If this is true, the length of the softening section and debonding section were substituted into Equation (13). If the result is true, the length of the softening section and debonding section are recorded as two arrays: *l_s_*__*record*_ and *l_d_*__*record*_. Then, a tiny increment Δ*l_s_* is added to the length of the softening section to obtain a new value. This new value is substituted into the judgement to check whether it is not larger than *l_s_*__*cri*2_. If it is true, this new lengths of the softening section and debonding section are substituted into Equation (13) again. If the obtained result is false, a tiny increment Δ*l_d_* is added to the length of the debonding section to obtain a new value. Then, this new value is substituted into Equation (13). These two loop functions are continued until the length of the softening section is larger than *l_s_*__*cri*2_.

**Figure 4:**
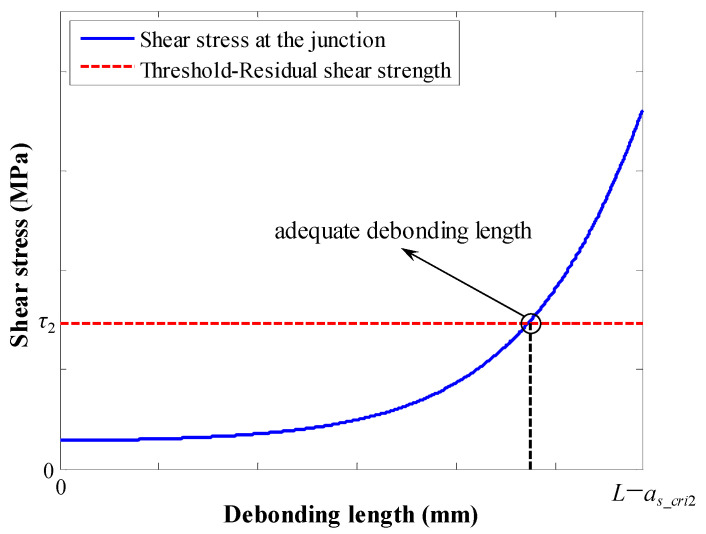
Variation trend of the SS at the junction between the softening section and debonding section when the length of the debonding section increases.

For a fixed length of the softening section, there is a specific length of debonding section. When the length of the debonding section increases, the variation trend of the SS at the junction point is shown in Figure 4. At the intersection between the blue line and red line, the corresponding length of the debonding section is the exact solution. However, this exact solution is quite complicated. Therefore, in this paper, for each length of the softening section, a tiny increment Δ*l_d_* is added to the length of the debonding section. When the SS at the junction is just beyond the residual bond strength, the corresponding length of the debonding section is regarded as the numerical solution.

**Figure 5:**
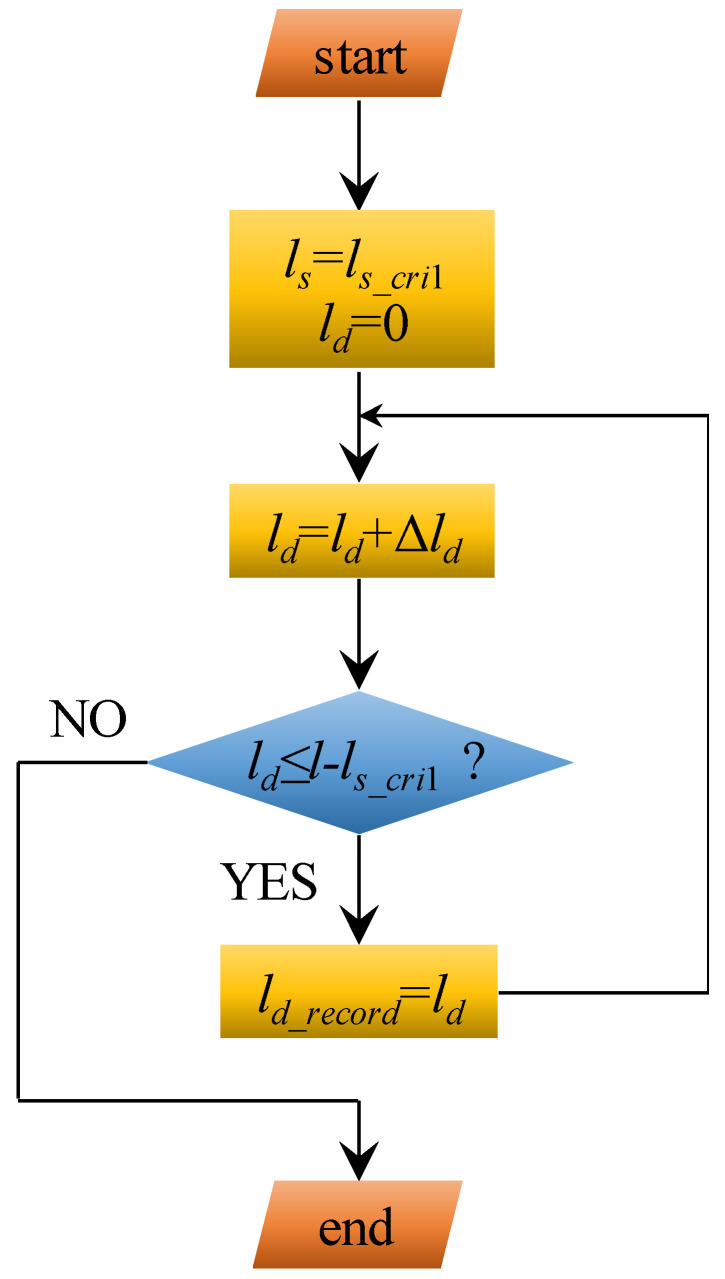
The enhanced calculation method to obtain the length of the softening section and debonding section.

When using this numerical calculation approach, a shortcoming is that two loop functions are needed. This requires massive iteration calculation. To simplify the calculation process, this paper proposes an enhanced calculation approach. The logic of this enhanced calculation method is shown in Figure 5. In this case, when the length of the softening section increases to the first critical value (*l_s_*__*cri*1_), it remains constant. Then, the length of the debonding section begins increasing from 0 to (*l*-*l_s_*__*cri*1_). During this increasing process of the debonding length, a tiny increment Δ*l_d_* is added to the length of the debonding section. Consequently, a new value is obtained. If this new value is not larger than (*l*-*l_s_*__*cri*1_), it is recorded into the array of *l_d_*__*record*_. Then, the tiny increment Δ*l_d_* continues to be added to the length of the debonding section. This loop function is terminated when the obtained length of the debonding section is larger than (*l*-*l_s_*__*cri*1_).

Compared with Figure 3, this enhanced calculation method only needs one loop. Therefore, the number of iteration calculations is much smaller. To realise the full calculation process, Matlab software R2012b was used.

## 4. Validation of the Numerical Calculation Method

To validate the credibility of the numerical calculation method, the experimental anchorage test conducted by Aoki et al. [36] was used as an example. During testing, a rock anchor whose diameter was 15.2 mm was used. The rock anchor was encapsulated in the artificial rock with an encapsulation length of 4 m. To simulate this anchorage test, the tri-linear law in Table 1 was used.

**Figure 6:**
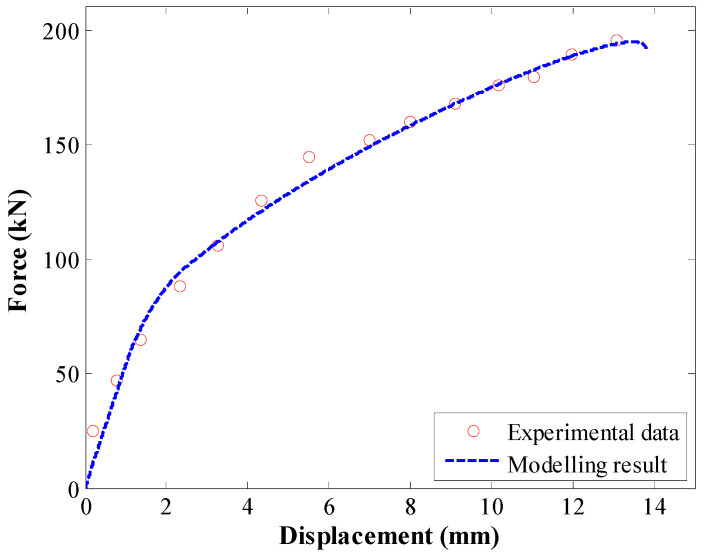
The first validation of the proposed calculation method.

Figure 6 shows the validation of the enhanced calculation method. A satisfactory correlation existed between experimental results and the enhanced calculation result. This validated that the proposed calculation method was credible.

To further validate this enhanced numerical calculation method, the experimental anchorage test conducted by Bai et al. [37] was modelled. Rock anchors had a radius of 14 mm and encapsulation length of 3 m. For the tri-linear law, the values in Table 2 were used.

**Figure 7:**
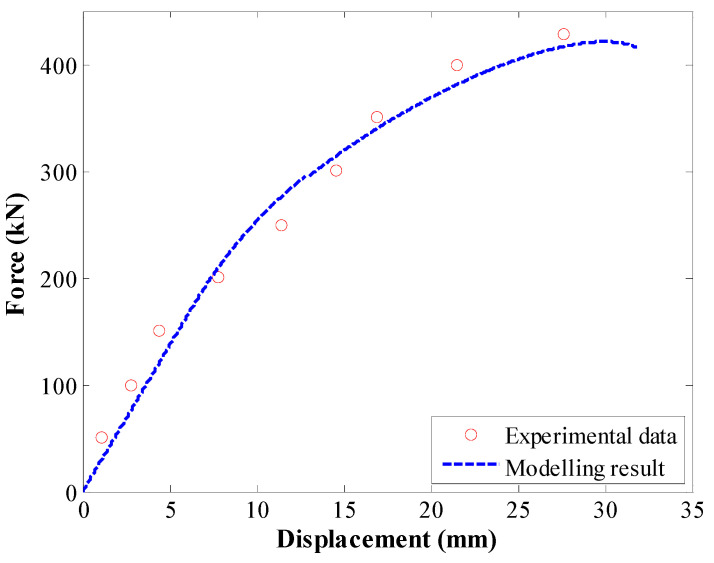
The second validation of the proposed calculation method.

The comparison between experimental tests and modelling results is shown in Figure 7. There was satisfactory agreement between experimental and modelling results, further confirming the accuracy of the proposed calculation method.

## 5. Case Studies

Case studies were further conducted to confirm the accuracy of this enhanced calculation method. Theoretical pull tests were conducted on rebars. The pull test was conducted following the method conducted by Li et al. [38]. A long rebar was installed in the confining medium. The grout was the bonding agent, connecting rebars with the confining medium. The external ends of the rebars were loaded along the upward direction. During the loading process, the pull load and displacement were recorded.

A rebar with a radius of 11 mm and Young’s modulus of 210 GPa was tested. The encapsulation length was 1 m. Previous experimental pull tests indicated that the rebar–grout interface showed apparent residual bond strength [34]. Referring to previous research [39], to evaluate the anchorage performance of rebars when the residual bond strength varied, two residual bond strengths, 1 MPa and 2 MPa, were used.

### 5.1. Pull Test When the Residual Bond Strength Equals 1 MPa

In the first series of tests, the input parameters of the tri-linear law are given in Table 3. These values in Table 3 are used to compare the anchorage performance of rebars calculated with the original calculation method and the enhanced calculation method.

**Figure 8:**
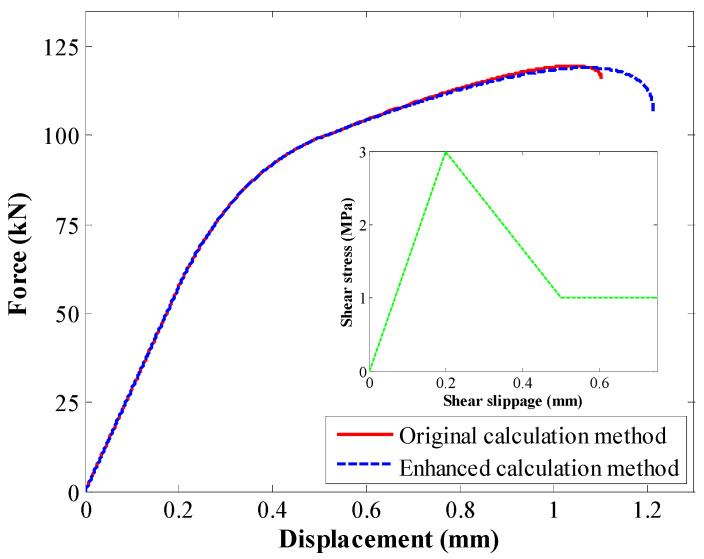
Comparison between the load–deformation relation in the first case study.

Two tests were conducted. In the first test, the original calculation method was used. In the second test, the enhanced calculation method was used. A comparison between the load–deformation relation is shown in Figure 8. Apparently, although the input tri-linear law was identical, the exported load–deformation relation was different. This difference was mainly reflected in the ESD stage. The maximum displacement in those two tests was apparently different. However, the overall trend of the load–deformation relation was consistent.

**Figure 9:**
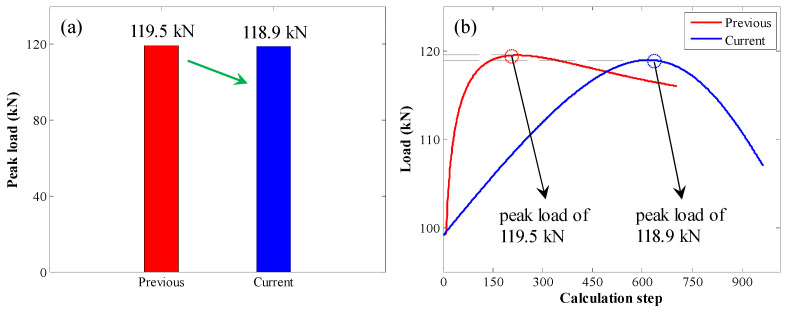
Comparison between peak loads: (**a**) bar chart of the peak load; (**b**) pull load variation trend with increasing calculation step.

The comparison between peak loads is shown in Figure 9. With the original calculation method, the peak load was 119.5 kN. With the enhanced calculation method, the peak load was 118.9 kN. There was no significant difference between them.

To further study the displacement performance of rebars when the two different calculation methods were used, the displacement at peak load and maximum displacement were analysed.

**Figure 10:**
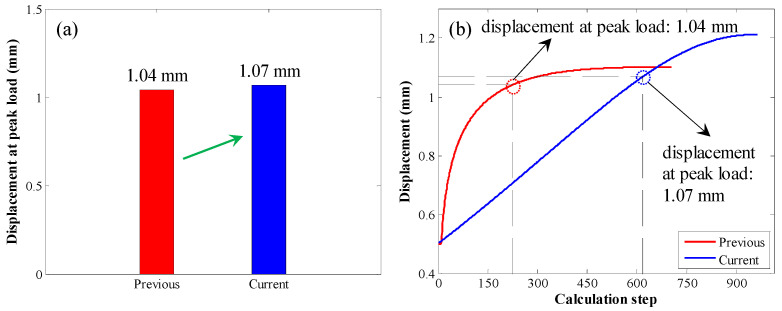
Comparison between the displacement at peak load in the first case: (**a**) bar chart of the displacement at peak load; (**b**) displacement variation trend with increasing calculation step.

For the peak load, the corresponding displacement is shown in Figure 10. When the original calculation method was used, the displacement at the peak load was 1.04 mm. When the enhanced calculation method was used, the displacement at the peak load was 1.07 mm. Therefore, with this enhanced calculation method, the displacement at the peak load was relatively larger.

**Figure 11:**
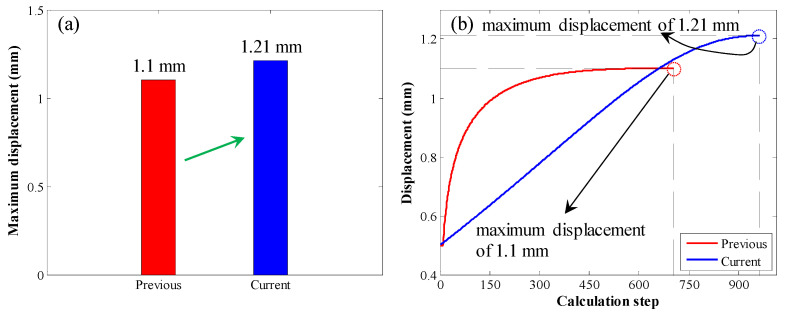
Comparison of maximum displacement in the first case: (**a**) bar chart of the maximum displacement; (**b**) displacement variation trend with increasing calculation step.

The maximum displacement was also compared, as shown in Figure 11. When the original calculation method was used, the maximum displacement was 1.1 mm. When the enhanced calculation method was used, the maximum displacement was 1.21 mm. Therefore, when the enhanced calculation method was used, the maximum displacement was relatively larger.

The above comparison shows that when the enhanced calculation method was used, the peak load of rebars was consistent with the original calculation method. By contrast, the displacement at the peak load and maximum displacement were relatively larger. Nevertheless, there was good agreement between the load–deformation relation obtained with these two calculation methods.

When the enhanced calculation method was used, the number of iteration calculations was significantly decreased. Therefore, the enhanced calculation method showed much better performance.

### 5.2. Pull Test When the Residual Bond Strength Equals 2 MPa

To further confirm the credibility of this enhanced calculation method, the other series of pull tests was conducted. During the calculation process, the residual bond strength of the rebar–grout interface was changed to 2 MPa. The specific input parameters of the rebar–grout interface are given in Table 4.

**Figure 12:**
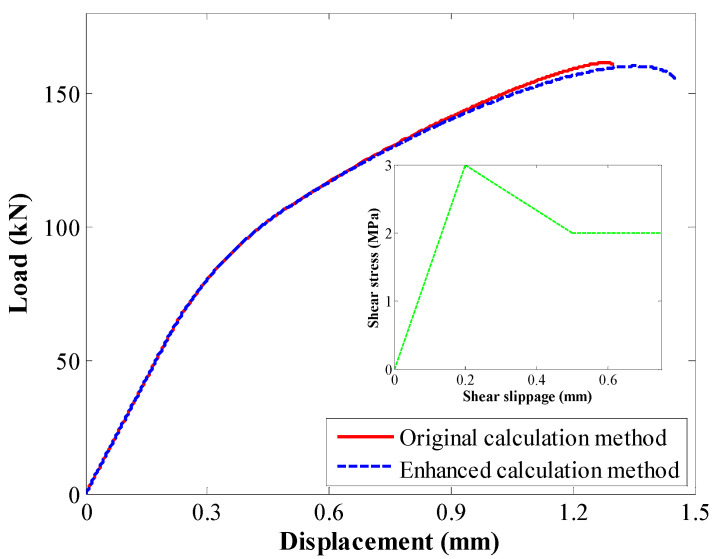
Comparison of the load–deformation relation in the second case study.

A comparison between the load–deformation relation results is shown in Figure 12. When the original calculation method and enhanced calculation method were used, there was a slight difference in the results. To better compare the obtained results, the peak load, displacement at peak load, and maximum displacement were compared.

**Figure 13:**
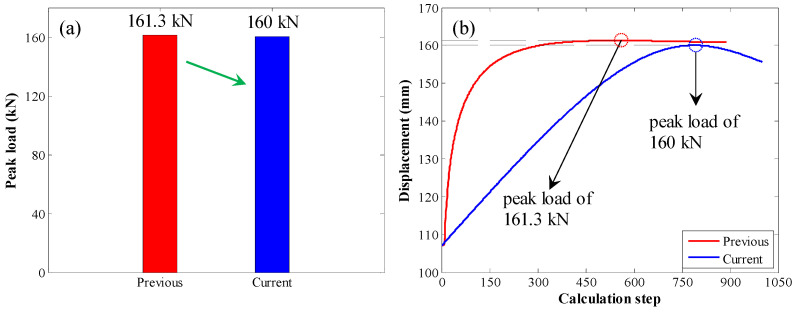
Comparison of the peak load: (**a**) bar chart of peak load; (**b**) pull load variation trend with increasing calculation step.

First, the peak load was compared, as shown in Figure 13. When the original calculation method was used, the peak load was 161.3 kN. When the enhanced calculation method was used, the peak load was 160 kN. Therefore, there was almost no apparent difference between them.

**Figure 14:**
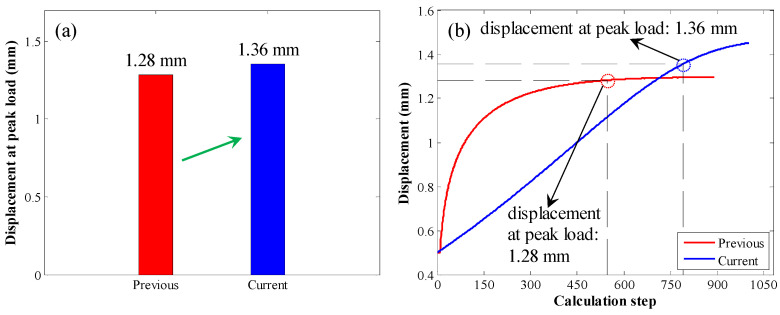
Comparison of the displacement at peak load in the second case: (**a**) bar chart of the displacement at peak load; (**b**) displacement variation trend with increasing calculation step.

The displacement at peak load was also compared, as shown in Figure 14. When the original calculation method was used, the displacement at peak load was 1.28 mm. By contrast, when the enhanced calculation method was used, the displacement at peak load was 1.36 mm. Therefore, when the enhanced calculation method was used, the displacement at peak load was relatively larger.

**Figure 15:**
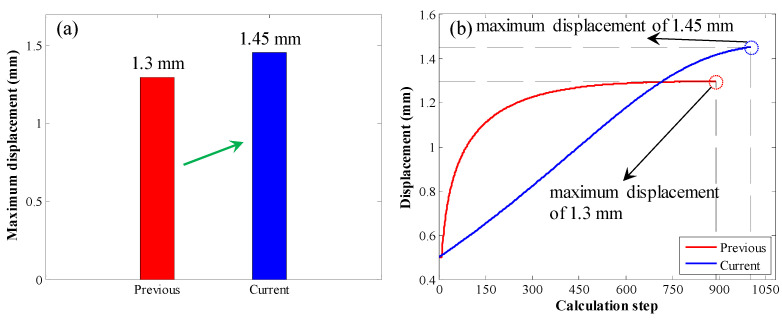
Comparison of the maximum displacement in the second case: (**a**) bar chart of the maximum displacement; (**b**) displacement variation trend with increasing calculation step.

Last, the maximum displacement was compared, as shown in Figure 15. When the original calculation method was used, the maximum displacement was 1.3 mm. When the enhanced calculation method was used, the maximum displacement was 1.45 mm. Therefore, compared with the original calculation method, when the enhanced calculation method was used, the maximum displacement was larger.

The above comparison shows a marginal difference between the load–deformation relation when these two calculation methods were used. This demonstrates the credibility of the enhanced calculation method. It is noted that when the enhanced calculation method was used, the number of iteration calculations was much smaller. Therefore, the enhanced calculation method showed much better performance in the calculation process.

## 6. Discussion

The SS at the rebar–grout interface provides resistance along the axial direction of rebars. To calculate the specific resistance, Ren et al. [35] proposed using the tri-linear law to depict the shear behaviour of the rebar–grout interface. However, the shortcoming was that when the rebar–grout interface entered the ESD stage, the calculation process was quite complicated. The reason was that in the ESD stage, the length of the softening section and debonding section mobilised simultaneously. This meant that the calculation efficiency was poor and not convenient for users to calculate the anchorage performance of rebars.

It is agreed that the current commercial software is sufficient in processing iterative calculations. However, to provide a simpler way for users to conveniently calculate the anchorage performance of rebars, this paper proposed an enhanced calculation method. This paper followed the modelling procedures proposed by Ren et al. [35]. However, this paper assumed that the length of the softening section was constant. Then, one loop function was created to calculate the length of the debonding section. Then, the length of the softening section was obtained. Based on this method, the number of iteration calculations was significantly decreased. This improved the calculation efficiency.

It is also noted that when the tri-linear law is used, the rebar–grout interface will experience the softening–debonding stage and debonding stage. Those two stages were not analysed in this paper. The reason is that previous research indicated that in the softening–debonding stage, there was a snapback phenomenon in the load–deformation relation [35]. This phenomenon was also monitored in other similar research [28]. However, this snapback phenomenon was not a true reflection of the uniaxial loading process of rebars. This is because during the uniaxial loading process of rebars, the tensile displacement of rebars increases monotonously. Nevertheless, the current remaining three stages can still reflect the anchorage performance of rebars.

In this study, the rock mass around rebars was treated as a homogeneous material. However, the in situ rock mass comprises joints and discontinuities. Therefore, the in situ rock mass is inhomogeneous. Based on the enhanced calculation method, further work can still be continued to study the anchorage performance of rebars when the rock mass is inhomogeneous.

## 7. Conclusions

This paper proposed an enhanced calculation method to calculate the load–deformation relation of rebars. The tri-linear law was used to illustrate the shear behaviour of the rebar–grout interface. The following conclusions were obtained.

1. When the rebar–grout interface entered the ESD stage, the length of the softening section was assumed to be constant. One loop function was created to calculate the length of the debonding section. This significantly reduced the number of iteration calculations.
2. Two independent experimental pull tests were used to validate the accuracy of this enhanced calculation method. There was a satisfactory correlation between experimental and numerical calculation results.
3. Two theoretical pull cases were conducted. The results obtained with the original calculation method and enhanced calculation method were compared. The peak load of rebars was similar. This indicated that the enhanced calculation method was sufficient in studying anchorage performance of rebars. However, the enhanced calculation method needs much fewer iteration calculations, which is more convenient and applicable.
4. When the enhanced calculation method was used, the displacement at peak load and the maximum displacement were relatively larger. However, those two displacements have no significant difference from the displacement obtained with the original calculation method.

## Figures and Tables

**Table 1:** The tri-linear law used in the first validation.

*τ*_1_ (MPa)	*u*_1_ (mm)	*τ*_2_ (MPa)	*u*_2_ (mm)
1.7	1	0.85	2.5

**Table 2:** The tri-linear law used in the second validation.

*τ*_1_ (MPa)	*u*_1_ (mm)	*τ*_2_ (MPa)	*u*_2_ (mm)
1.8	6.5	1.5	13

**Table 3:** Tri-linear law used in the first case study.

*τ*_1_ (MPa)	*u*_1_ (mm)	*τ*_2_ (MPa)	*u*_2_ (mm)
3	0.2	1	0.5

**Table 4:** Tri-linear law used in the second case study.

*τ*_1_ (MPa)	*u*_1_ (mm)	*τ*_2_ (MPa)	*u*_2_ (mm)
3	0.2	2	0.5

## Data Availability

This article includes all data.

## Nomenclature and Abbreviations

*A*	cross-section area of the rebar
*b*	constant coefficient
*c*	perimeter of the rebar
*E*	Young’s modulus of the rebar
*k*	slope between the shear stress (SS) and shear slipping
*l*	encapsulating length of the rebar
*l_s_*	length of the softening section
*l_d_*	length of the debonding section
*p*	tensile load of the rebar
*r*	radius of the rebar
*s*	shear slipping of the rebar–grout interface
*u*	tensile displacement of the rebar
*u*_1_	shear slipping when the bond strength is reached
*u*_2_	shear slipping when the residual bond strength is reached
*α*	variable coefficient
*τ*	SS at the rebar–grout interface
*τ*_1_	bond strength of the rebar–grout interface
*τ*_2_	residual bond strength of the rebar–grout interface
*ω*	variable coefficient
